# A preoperative magnetic resonance imaging can aid in staging and treatment choice for upper tract urothelial carcinoma

**DOI:** 10.1002/bco2.337

**Published:** 2024-02-24

**Authors:** Bjarte Almås, Lars Anders Rokne Reisæter, Carl Erik Markhus, Karin Margrethe Hjelle, Astrid Børretzen, Christian Beisland

**Affiliations:** ^1^ Department of Urology Haukeland University Hospital Bergen Norway; ^2^ Department of Clinical Medicine University of Bergen Bergen Norway; ^3^ Department of Radiology Haukeland University Hospital Bergen Norway; ^4^ Department of Pathology Haukeland University Hospital Bergen Norway

**Keywords:** diagnosis, MRI, prospective study, staging, upper tract urothelial carcinoma

## Abstract

**Objectives:**

The aim was to investigate the predictive abilities of a preoperative diffusion‐weighted MRI (dwMRI) among patients with surgically treated upper tract urothelial carcinoma (UTUC).

**Materials and methods:**

Written consent was obtained from all participants in this prospective and ethically approved study. Thirty‐five UTUC patients treated with radical surgery were examined with a preoperative dwMRI and prospectively included during 2017–2022. Two radiologists examined the CT scans and dwMRIs for radiological stage, and the apparent diffusion coefficient (ADC) in the tumours at the dwMRI was registered. The radiologists were blinded for patient history, final histopathology and the readings of the other radiologist. The radiological variables were analysed regarding their abilities to predict muscle‐invasive disease (MID, T2–T4) and tumour grade at final pathology after radical surgery. The predictive abilities were assessed using chi‐square tests, Student's *t*‐test and calculating the area under the curve in a receiver operating characteristic (ROC) curve. Correlation between the two radiologists was quantified calculating the intra‐class correlation coefficient. *P*‐values <0.05 were considered statistically significant.

**Results:**

Mean age was 72 years, 20 had high‐grade tumour, and 13 patients had MID. The ADC values at the dwMRI were significantly lower among patients with MID compared to patients with non‐muscle‐invasive disease (930 vs 1189, *p* = <0.001). The area under the ROC curve (AUC) in an ROC curve to predict MID was 0.88 (CI 0.77–0.99, *p* = <0.001). The ADC values were significantly lower among patients with high‐grade tumours compared to low‐grade tumours (1005 vs 1210, *p* = 0.002). The correlation of the ADC measurements between the two radiologists was of 0.93 (CI 0.85–0.96, *p* < 0.001).

**Conclusion:**

Tumour ADC at the MRI emerges as a potential biomarker for aggressive disease. The results are promising but should be validated in a larger, multicentre study.

## 1 INTRODUCTION

The incidence of upper tract urothelial carcinoma (UTUC) is increasing in Europe, with a current incidence of 4–5: 100 000.^1^, ^2^ Advanced cases of UTUC have a poor prognosis; the 5‐year survival in case of locally advanced disease is referred to be about 50%.^3^ Standard treatment currently consists of radical surgery most commonly as a radical nephroureterectomy (RNU), with the possibility of giving adjuvant chemotherapy or immunotherapy in case of muscle‐invasive disease (MID).^4^, ^5^, ^6^ Due to the severity of the disease, intensified therapy such as extended lymph node dissection and neo‐adjuvant chemotherapy have been suggested for high‐risk disease.^7^ The indications for these treatments are not yet fully established. One reason for this is that the correct preoperative staging of UTUC is challenging with the current available diagnostic work‐up. Standard work‐up according to current guidelines consists of a CT urography (CTU), with the possibility of a supplementary ureteroscopy (URS) if the diagnosis is unclear or if the patient is a candidate for nephron‐sparing surgery such as endoscopic ablation or ureteral resection.^4^

Small, retrospective single‐centre studies have indicated the potential benefit of a magnetic resonance imaging (MRI) in the preoperative evaluation of UTUC, especially the use of the apparent diffusion coefficient (ADC) in the tumour measured using a diffusion‐weighted MRI (dwMRI).^8^, ^9^ Current guidelines recommend the use of MRI only if CTU is contraindicated.^4^

The primary aim of the present study was to assess the predictive abilities of a dwMRI in the preoperative staging of UTUC, both as an isolated procedure and in addition to the CTU. The secondary aim was to assess the interobserver correlation between two different radiologists when interpreting a dwMRI with this indication.

## 2 METHOD

After obtaining approval from the regional ethics committee (ref no. 2017/931), informed consent was obtained from potential participants in this prospective study. Inclusion criteria were patients with a probable UTUC >10 mm as diagnosed at a CTU with or without the addition of a diagnostic URS with a biopsy. The patients then proceeded to a preoperative MRI before radical surgery. A total of 39 patients were examined with a dwMRI in the study during 2017–2022. Four were later excluded due to non‐UTUC histology leaving 35 patients for final inclusion.

All relevant information about the patients, radiological examinations, tumours and treatment was gathered and entered into a database. Two independent radiologists (LAR and CEM) assessed the CTUs and the dwMRI scans. The radiological stage for both the CTU and the dwMRI was evaluated together with the ADC values of the tumours at the dwMRI scans. The ADC values were measured in regions of interest in the tumours at the radiologists' s discretion. The radiologists examined the dwMRIs and identified subjectively the areas within the tumour with a homogenous appearance most suitable for ADC measurement. The average ADC values between the two radiologists were calculated and used for purpose of analyses. The radiologists were blinded for patient history, final histology and the readings of the other radiologist. One of these radiologists (LAR) had long experience in uro‐radiology and measurement of ADC values at a dwMRI of the prostate, while the other radiologist (CEM) was an experienced uro‐radiologist, with less experience from ADC measurements at a dwMRI. To minimise recall bias as much as possible, the radiologists were given patient lists in randomised orders, and the CTUs and dwMRIs were examined separately with at least 2 weeks between the examinations.

## 3 RADIOLOGICAL METHODS

The CTU was performed using various CT scanners and with a standardised CTU protocol with non‐contrast and multiple contrast phases (arterial, portovenous and excretory). A 3‐T Siemens Skyra scanner was used to acquire the dwMRIs. A simplified MRI protocol that lasted less than 30 min without contrast enabled examinations independent of kidney function. Three coronal acquisitions, [T1 (FS), T2 and T2 space] with 5 mm thickness and two transversal acquisitions (T1 in and out of phase and with 3 mm slice thickness were used). Diffusion‐weighted images with both three and seven b‐values (50–1600) were analysed separately, to assess if there was a difference in the predictive ability with different dwMRI protocols. The findings according to three b‐values were abbreviated 3bADC, and those using seven b‐values were abbreviated 7bADC.

## 4 STATISTICAL METHODS

The predictive abilities of the CTUs and MRIs were assessed by comparing the radiological variables to the final pathology after radical surgery. Tumour stage and grade were chosen as outcome measures. Specifically, the presence of MID (pT2–T4 or pN+) as compared to non‐muscle‐invasive disease (NMID, pTa–T1 and N0/Nx) and tumour grade according to the 2004 WHO classification (low grade or high grade) were chosen as outcome variables.^10^ Categorical variables were assessed using the chi‐square method and continuous variables using Student's *t*‐test. The predictive abilities were further explored by calculating the area under the curve (AUC) in a receiver operating characteristic (ROC) curve. The correlation between the two radiologists' interpretations was quantified by calculating the intra‐class correlation coefficient using a two‐way mixed model of the absolute agreement type. SPSS version 29 was used. *P*‐values <0.05 were considered statistically significant.

## 5 RESULTS

Patient, treatment and tumour characteristics are displayed in Table 1. The median age of the patients was 72 (IQR 65–78) years, and eight of 35 patients were women. Haematuria was the presenting symptom among 24 patients, and 22 were ECOG 0. Of the tumours, 23 were located in the renal pelvis, 20 were high grade, and 13 had MID. Of the patients, 33 were treated with an RNU, one with an RNU as part of a cystoprostatectomy and one with a radical distal segmental ureteral resection. The patients were treated with an open (*n* = 14), laparoscopic (*n* = 9) or robot‐assisted (*n* = 12) procedure.

**TABLE 1:** Patient and tumour characteristics.

		% of total
Age years mean (median, IQR)	72 (72, 65–78)	
Gender	No.	
Male	27	77
Female	8	23
Symptoms		
Haematuria	24	69
None	7	20
Flank pain or general symptoms	4	11
ECOG		
0	22	63
1+	13	37
Smoking status		
Current smoker	11	31
Previous smoker	14	40
Never smoked	10	29
Diagnostic ureterrenoscopy		
Yes	21	60
No	14	40
Tumour primary location		
Renal pelvis	23	66
Ureter	12	34
Tumour grade^a^		
Low	15	43
High	20	57
Muscle‐invasive disease^b^		
Yes	13	37
No	22	63
Type of surgery		
Open	14	40
Laparoscopic	9	26
Robot assisted	12	34
Template‐based lymph node dissection		
Yes	12	34
No	23	66

Abbreviation: ECOG, Eastern Cooperative Oncology Group.

^a^ Tumour grade according to WHO/ISUP classification 2004.

^b^ pT2–T4 and/or pN+ disease at final pathology after nephroureterectomy.

Radiological stage or tumour size did not predict MID in the current cohort. The following results are presented as the *average* ADC measurements between the two radiologists using the simplified model with three b‐values for ADC measurement (3bADC). For detailed results regarding the different ADC measurements of both radiologists and both ADC measurement methods, see Tables 2 and 3.

**TABLE 2:** Demonstrates the findings of the two radiologists regarding tumour stage for both the CTU and the dwMRI.

	Muscle‐invasive disease^a^	Non‐muscle‐invasive disease^b^	ICC^c^	*p*
Radiological variables				
CTU size (mm)				
Radiologist 1	46 mm	46 mm		1
Radiologist 2	43 mm	45 mm		0.9
CTU stage				
Radiologist 1	3/13^d^	18/22^d^		0.7
Radiologist 2	6/13^d^	16/22^d^		0.1
MRI stage				
Radiologist 1	4/13^d^	20/22^d^		0.1
Radiologist 2	4/13^d^	19/22^d^		0.2
dwMRI 3bADC^e^				
Radiologist 1	936	1206		<0.001
Radiologist 2	923	1173		<0.001
Average between radiologists	930	1189		<0.001
Interobserver correlation			0.93	<0.001
dwMRI 7bADC^f^				
Radiologist 1	948	1194		<0.001
Radiologist 2	903	1173		<0.001
Average between radiologists	926	1184		<0.001
Interobserver correlation			0.83	<0.001

^a^ Muscle‐invasive disease was defined as pT2–T4 disease.

^b^ Non‐muscle‐invasive disease was defined as pTa–T1 and pN0/Nx.

^c^ Intra‐class correlation coefficient between the two radiologists.

^d^ Correctly staged by radiologist.

^e^ apparent diffusion coefficient (ADC) values in tumours using simplified measurement method with 3 b‐values.

^f^ ADC values in tumours using full measurement method with 7 b‐values.

**TABLE 3:** Demonstrates the results of the radiologists regarding tumour grade.

Histology tumour grade	High‐grade tumour^a^	Low‐grade tumour	*p*‐value
dwMRI 3bADC^b^			
Radiologist 1	1013	1229	0.005
Radiologist 2	998	1190	0.002
Average dwMRI 3bADC3	1005	1210	0.002
dwMRI 7bADC^c^			
Radiologist 1	1018	1216	0.008
Radiologist 2	996	1174	0.008
Average dwMRI 7bADC	1007	1195	0.004

^a^ Tumour grade according to the 2004 WHO classification (high/low‐grade).

^b^ Apparent diffusion coefficient (ADC) values in tumours using simplified measurement method with 3 b‐values.

^c^ ADC values in tumours using full measurement method with seven b‐values.

**FIGURE 1:**
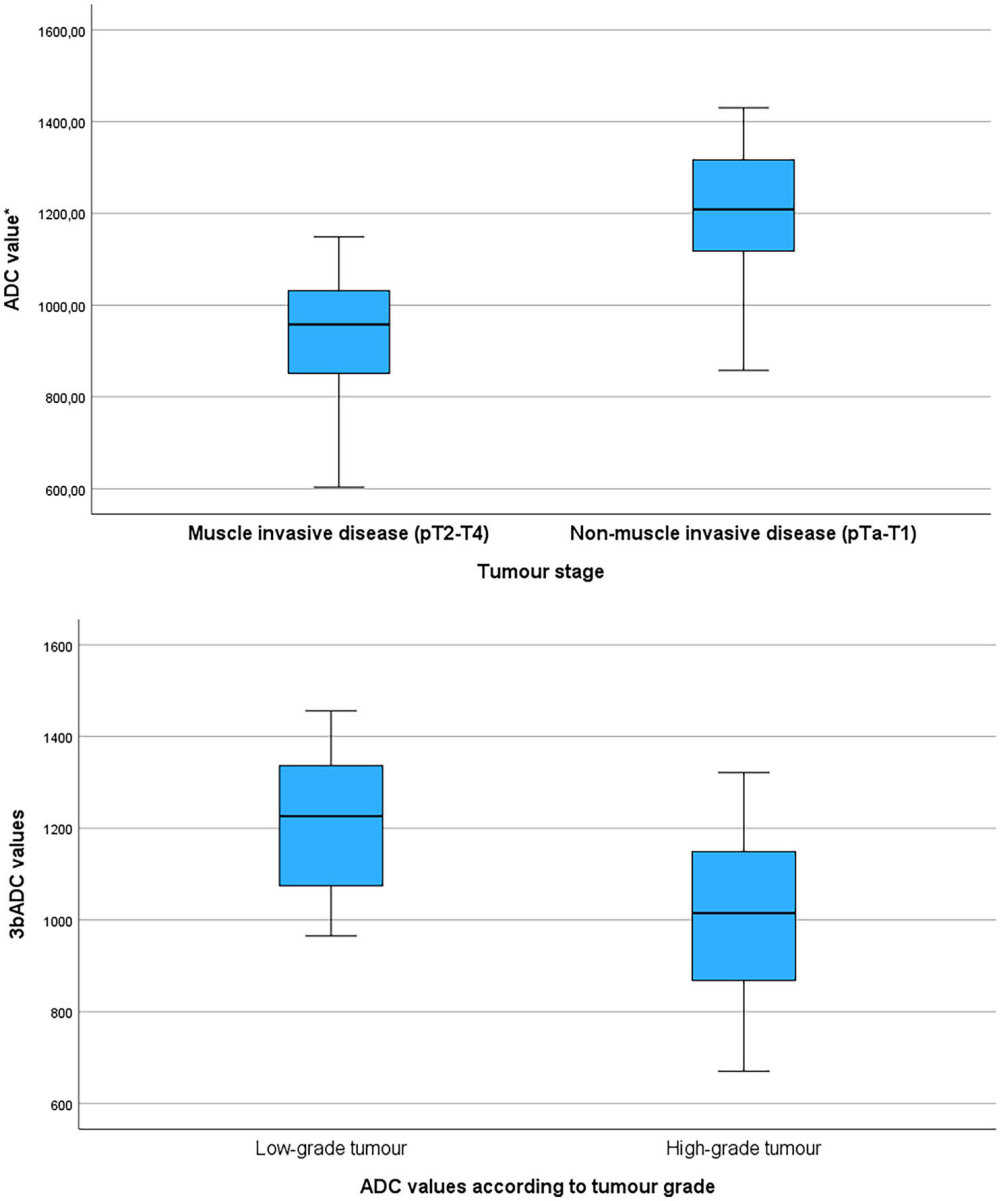
(A) The box plot illustrates the apparent diffusion coefficient (ADC) values in the tumours according to tumour stage. The ADC values presented are the average values of the two radiologists using the simplified 3bADC model. Muscle‐invasive disease was defined as pT2–T4 or pN+ at final histology. Non‐muscle‐invasive disease was defined as pTa–T1 and N0/Nx. The ADC values were significantly lower patients with non‐organ confined disease compared to patients with organ‐confined disease (mean 930 vs 1189, *p* = <0.001). (B) The box plot illustrates the apparent diffusion coefficient (ADC) values in the tumours according to tumour grade as defined in the 2004 WHO classification into high‐/low‐grade tumours. The ADC values presented are the average values of the two radiologists using the simplified 3bADC model. The ADC values were significantly lower among patients with high‐grade tumours compared to low‐grade tumours (1005 vs 1210, *p* = 0.002).

**FIGURE 2:**
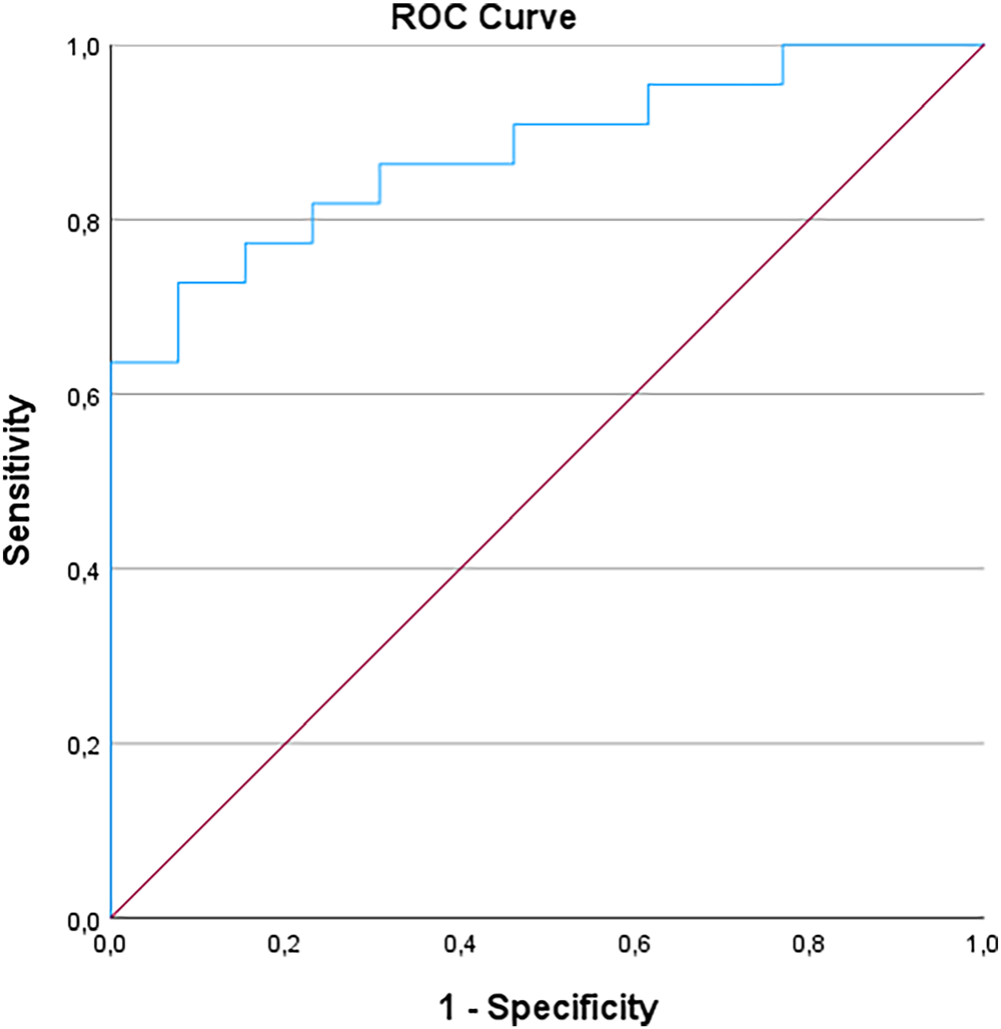
The receiver operating characteristics (ROC) curve illustrates the predictive ability of the apparent diffusion coefficient (ADC) value in the tumours to predict muscle invasive disease at final pathology after radical surgery for UTUC. The average ADC values of the two radiologists using the simplified model with three b‐values were chosen in the illustration. The area under the curve was 0.88 (CI 0.77–0.99, *p* < 0.001).

The ADC values were significantly lower among patients with MID compared to patients with NMID (930 vs 1189, *p* = <0.001) (see Figure 1). The AUC in a ROC curve to predict NOCD was 0.88 (CI 0.77–0.99, *p* = <0.001) (see Figure 2). Analyses of the ROC curve showed that using an ADC cut‐off value of 1050 discriminated best between MID and NMID tumours. An ADC < 1050 predicted MID with a sensitivity and specificity of 77% and 82%, respectively.

The ADC values were significantly lower among patients with high‐grade tumours compared to low‐grade tumours (1005 vs 1210, *p* = 0.002) (see Figure 1). An ADC > 1200 predicted a low‐grade tumour with a sensitivity and specificity of 53% and 90%, respectively.

The correlation of the 3bADC measurements between the two readers is demonstrated in Figure S1. Although some outliers exist, the correlation is in general high, quantified with an intra‐class correlation coefficient of 0.93 (CI 0.85–0.96, *p* < 0.001). The intra‐class correlation coefficient regarding the full (7bADC) model was 0.83 (CI 0.66–0.91, *p* < 0.001).

## 6 DISCUSSION

The main finding in the present study was that the ADC in a dwMRI was a strong predictor of both tumour stage and grade at final pathology after radical surgery for UTUC. The findings were consistent between two independent radiologists.

The predictive abilities of the ADC values at a dwMRI have previously been demonstrated for a variety of both benign and malign conditions. It is in clinical use in the diagnosis of ischaemic stroke, non‐urological cancers and prostate cancer.^11^, ^12^, ^13^ Regarding UTUC, the potential use of ADC at a dwMRI as a biomarker for aggressive disease has been demonstrated in several smaller, retrospective single‐centre studies previously.

The ability of the ADC to predict tumour grade (high grade vs low grade) in UTUC has been described in two retrospective studies,^8^, ^9^ while two other studies have described that the ADC can predict both metastatic potential and survival.^14^, ^15^ Despite these findings, the use of dwMRI in the preoperative setting for UTUC is limited. There is a paucity of newer, multicentre and prospective studies on the subject.

To our knowledge, this is the first prospective European study on the use of a dwMRI in the preoperative staging of UTUC. One problem with identifying a continuous variable as a predictor for favourable or unfavourable tumour characteristics is the difficulty in setting a clinically useful cut‐off value. In the present study, we used the ROC curve analyses and identified an ADC cut‐off value of 1050 as the most useful in determining tumour stage. An ADC < 1050 predicted MID with a sensitivity and specificity of 77% and 82%, respectively. This is potentially clinically useful. If a patient who is otherwise fit to receive chemotherapy is diagnosed with a UTUC at the CTU, but the CTU is inconclusive regarding tumour stage, a dwMRI can be done as a supplementary examination. If the ADC in the tumour is low (in the present study <1050), this is suggestive of aggressive disease and this information can be used in the decision‐making regarding lymph node dissection and/or the use of neo‐adjuvant chemotherapy. There is also a potential use regarding low‐grade UTUC that can be candidates for nephron‐sparing surgery such as endoscopic laser ablation or segmental ureteral resection. If the CTU diagnoses a UTUC that might be a candidate for nephron sparing surgery, a high ADC (in the present study >1200) in the tumour is suggestive of a low‐grade disease that might be suitable for these treatment modalities. A diagnostic URS with a biopsy can of course yield information about tumour grade and feasibility of endoscopic treatment, but this procedure costs time and resources, the sampling error of URS biopsy is well known,^16^ and there is the described increased risk of later bladder recurrence after RNU among patients examined with a preoperative URS.^17^, ^18^ Thus, a preoperative dwMRI can be a useful option also among patients considered for nephron‐sparing surgery.

Despite the findings in this study, one should be very cautious to draw too firm conclusions about the clinical utility and potential cut‐off values for ADC based on this study alone. This is a single‐centre study with a limited number of patients. In addition, we know that the ADC value can vary between different MRI scanners and our absolute values are thus not directly transferrable to the findings on other MRI scanners. The findings should be validated in a larger, multicentre study. The specific cut‐off values would have to be determined specifically for each centre before taken into clinical practice. In a potential multicentre study on this subject, this difference between findings on different scanners could be overcome by calculating z‐values and performing analyses according to them instead of on the ADC values alone.

We found a high interobserver correlation between the two radiologists regarding all aspects of the readings quantified with an intra‐class correlation coefficient of 0.93 regarding the 3bADC measurements. This is interesting since only one of them had previous high experience in ADC measurements. Our findings indicate that reliable ADC measurements can be made even if the radiologist is not very proficient measuring ADC values from previous practice. The predictive abilities of the ADC values of both the simplified (3bADC) and comprehensive (7bADC) were confirmed, but no model was found to be superior to the other. This indicates that a quite simplified MRI protocol without contrast and using only three b‐values could be sufficient for stage and grade predictions. A simplified MRI protocol like this would last approximately 25 min with the scanner used in the present study.

The inability of radiological stage to predict final pathology in the present study might seem surprising. The ability of CTU in setting the correct diagnosis regarding UTUC is excellent,^19^ and the ability of CTU to predict pathological stage has also been demonstrated in several publications,^20^, ^21^ though the accuracy in these predictions is lower than ideal. The present study is a single‐centre study with a limited number of patients where the staging according to dwMRI was the primary aim. We attribute the inability of the radiological stage to predict the tumour stage in the present study to a relatively low patient number and that the study is underpowered to detect the true predictive abilities of the radiological stage of the CTU and dwMRI.

### 6.1 Strengths and limitations

The main strength of the present study is its prospective design with clearly defined a priori aims according to protocol. Power analyses were performed to predict the number of patients needed to answer the main research questions. Two independent radiologists with different backgrounds blinded for final pathology and patient data examined the CTUs and dwMRIs to increase the generalisability of our findings as much as possible. The study's main limitation is a relatively low patient number and its single‐centre design, and the findings should be confirmed in a larger, multicentre study.

## 7 CONCLUSION

The ADC at a dwMRI was shown to be a predictor of both tumour stage and grade in this prospective study. The findings were consistent between two independent radiologists and our findings indicate that a simplified dwMRI protocol can be used.

## AUTHOR CONTRIBUTIONS

**Bjarte Almås:** Conceptualization; investigation; data curation; statistical analysis; writing—original draft; writing—review and editing. **Lars Anders Rokne Reisæter:** Conceptualization; investigation; data curation; writing—review and editing. **Carl Erik Markhus:** Investigation; data curation; writing—review and editing. **Karin Margrethe Hjelle:** Data curation; writing—review and editing. **Astrid Børretzen:** Data curation; writing—review and editing. **Christian Beisland:** Conceptualization; statistical analysis; writing—review and editing.

## CONFLICT OF INTEREST STATEMENT

None.

## Supporting information


**Figure S1.** Bland–Altmann plot demonstrating the correlation between the two radiologists' interpretation of the apparent diffusion coefficient (ADC) values using the simplified measurement method with 3 b‐values. Even though some outliers exist, the correlation is in general high, quantified with an intraclass correlation coefficient of 0.93. The red dotted line illustrates the mean difference between the readers, while the green dotted lines illustrate the confidence intervals of the difference between the readers.
**Data S1.** Pre submission checklist
