# Supplementary figures and images for: A preoperative magnetic resonance imaging can aid in staging and treatment choice for upper tract urothelial carcinoma
Source: BJUI Compass. 2024 Feb 24;5(5):476–82. doi: 10.1002/bco2.337 (PMC11090765; doi:10.1002/bco2.337)

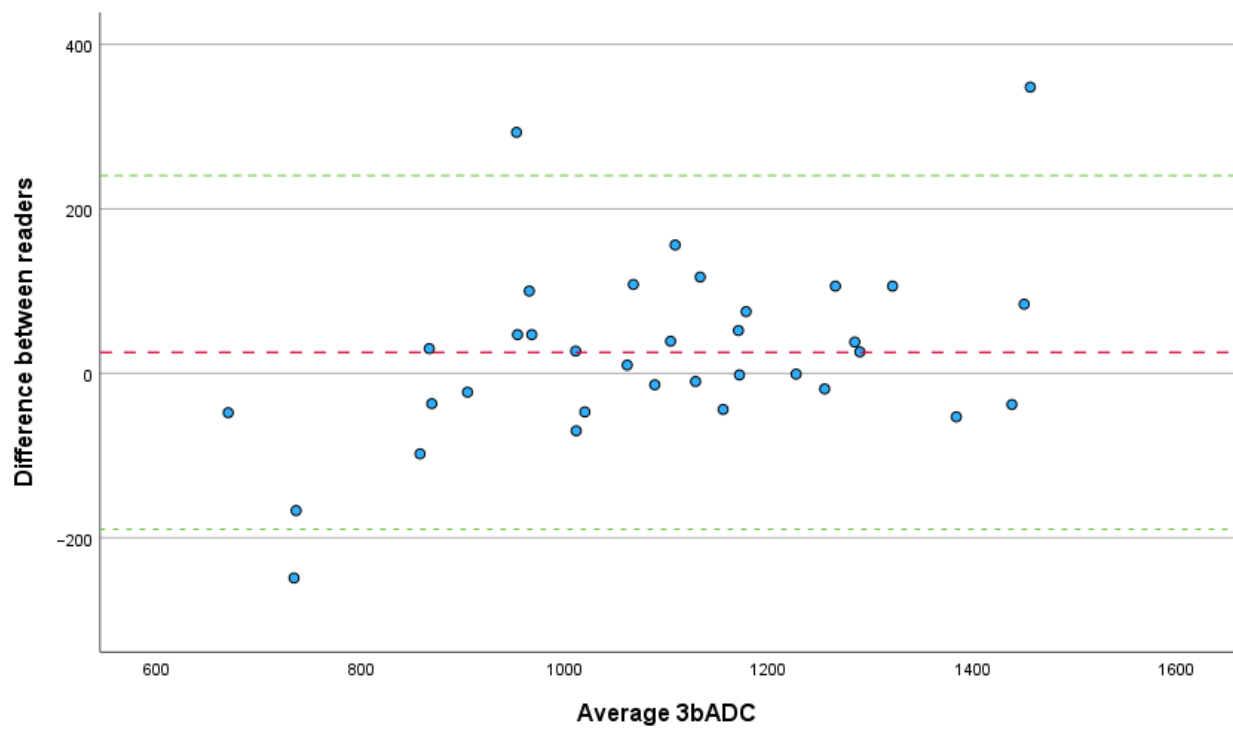

Supplement: Supplementary file 1 — Figure S1. Bland–Altmann plot demonstrating the correlation between the two radiologists' interpretation of the apparent diffusion coefficient (ADC) values using the simplified measurement method with 3 b‐values. Even though some outliers exist, the correlation is in general high, quantified with an intraclass correlation coefficient of 0.93. The red dotted line illustrates the mean difference between the readers, while the green dotted lines illustrate the confidence intervals of the difference between the readers. Data S1. Pre submission checklist [file BCO2-5-476-s001.pdf]
